# Supplementary figures and images for: Mechanistic molecular responses of the giant clam Tridacna crocea to Vibrio coralliilyticus challenge
Source: PLoS One. 2020 Apr 10;15(4):e0231399. doi: 10.1371/journal.pone.0231399 (PMC7148125; doi:10.1371/journal.pone.0231399)

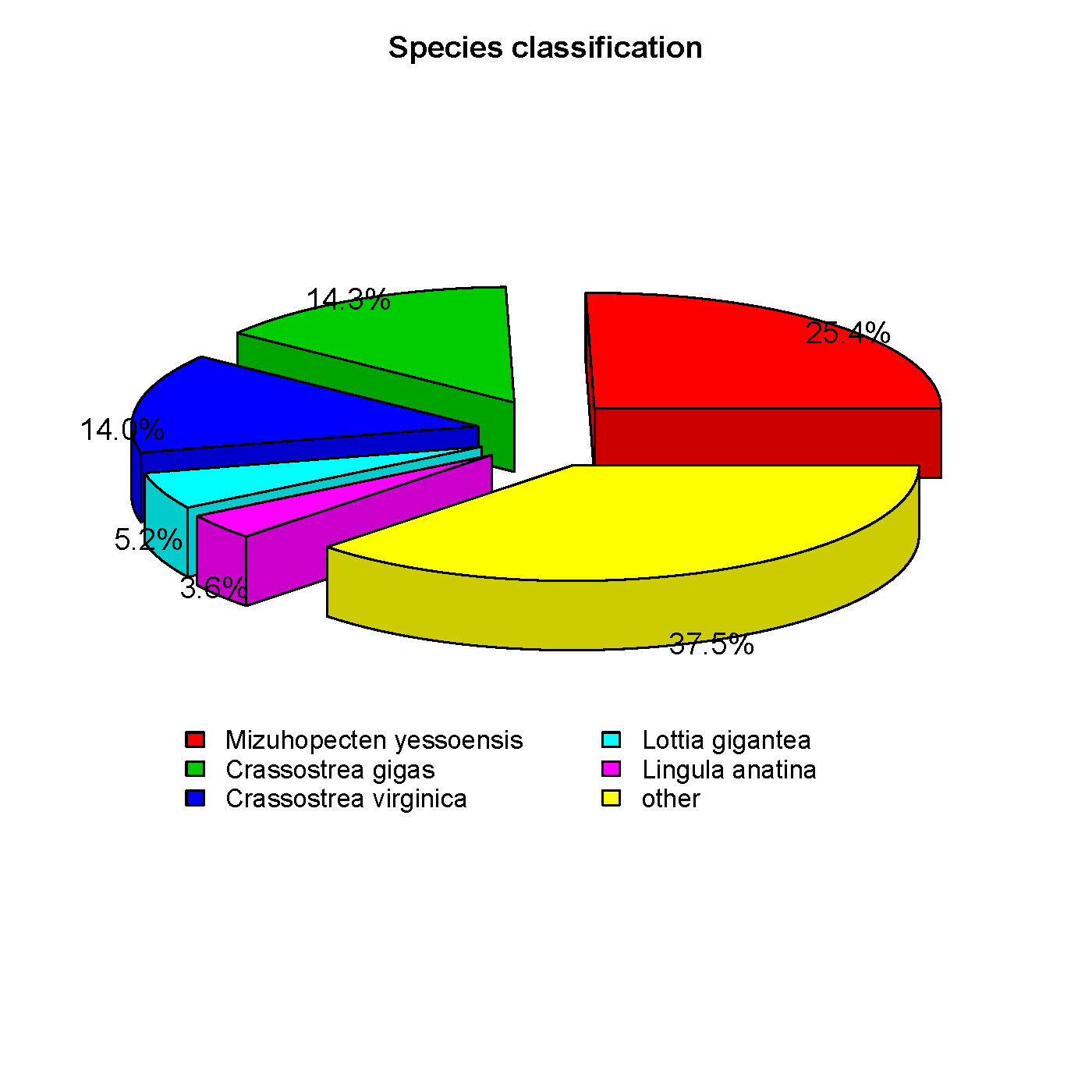

Supplement: S1 Fig — This figure shows the species distribution of unigene BLASTX matches against the NR protein database with a cut-off value E < 10−5 and the proportions for each species. Different colors represent different species. (TIF) [file pone.0231399.s001.tif]

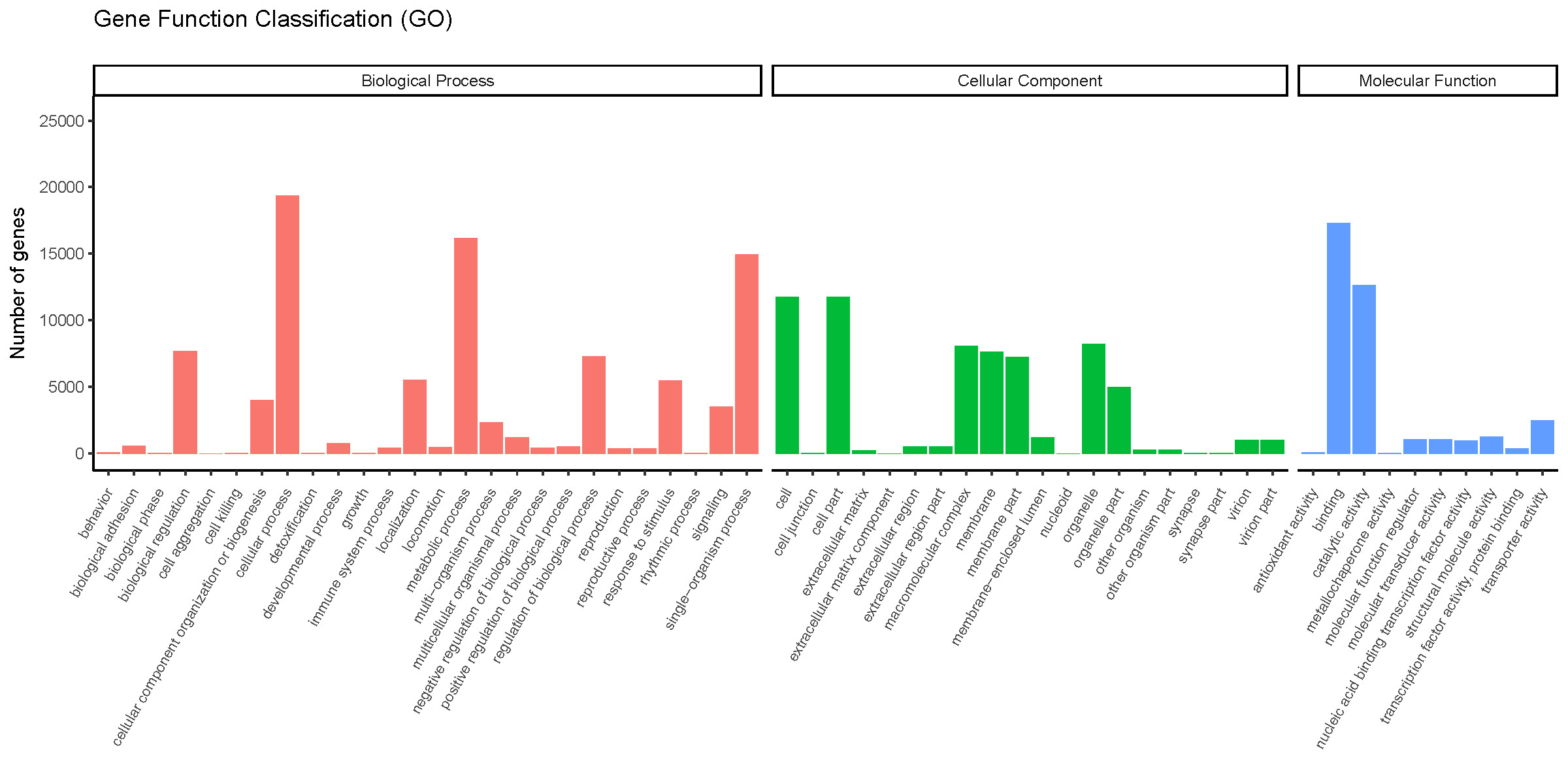

Supplement: S2 Fig — Unigenes were annotated in three categories: biological processes (red), cellular components (green), molecular functions (blue). Each bar represents the relative abundance of unigenes classified under each category at level 2. (TIF) [file pone.0231399.s002.tif]

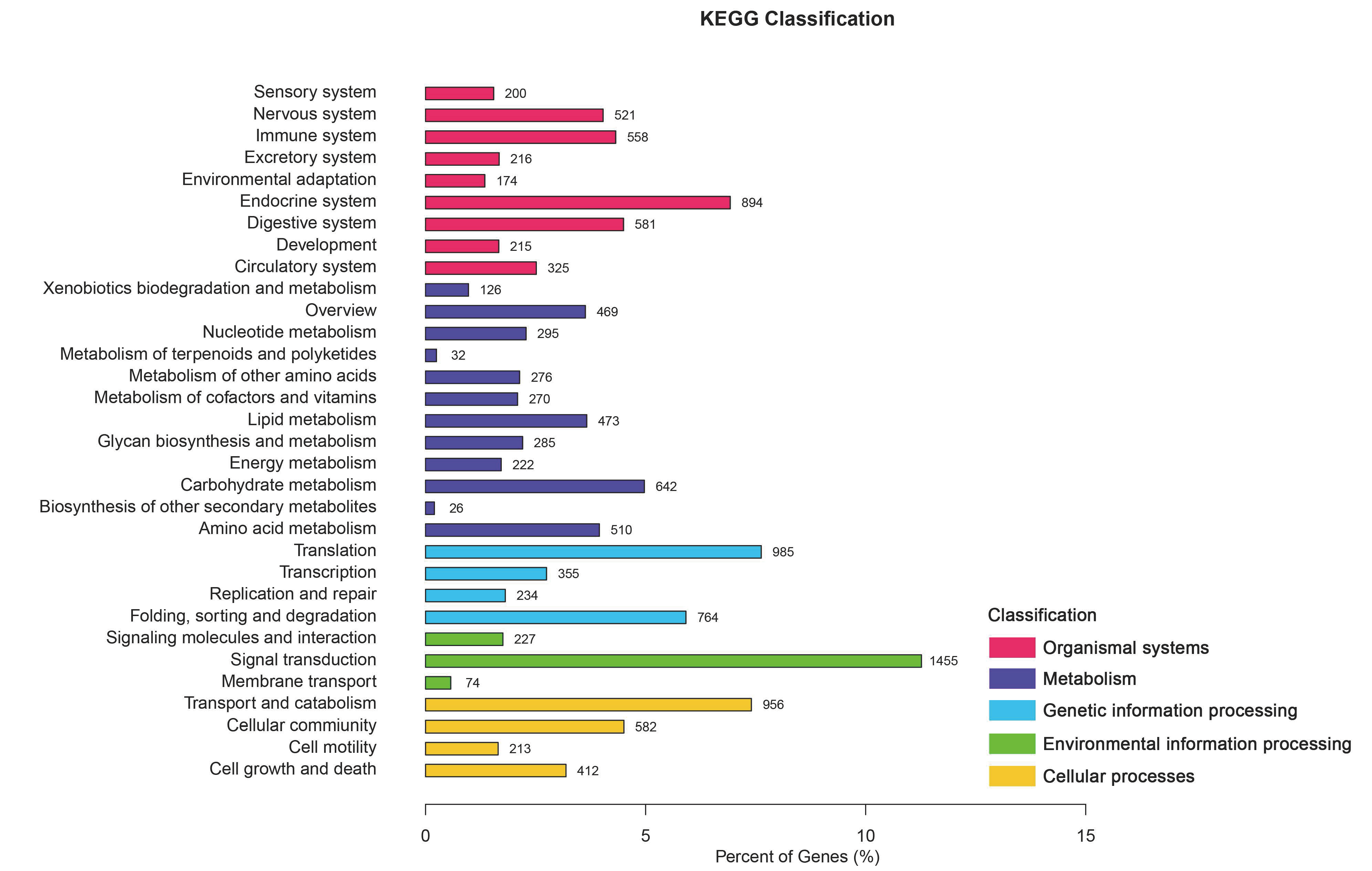

Supplement: S3 Fig — (TIF) [file pone.0231399.s003.tif]

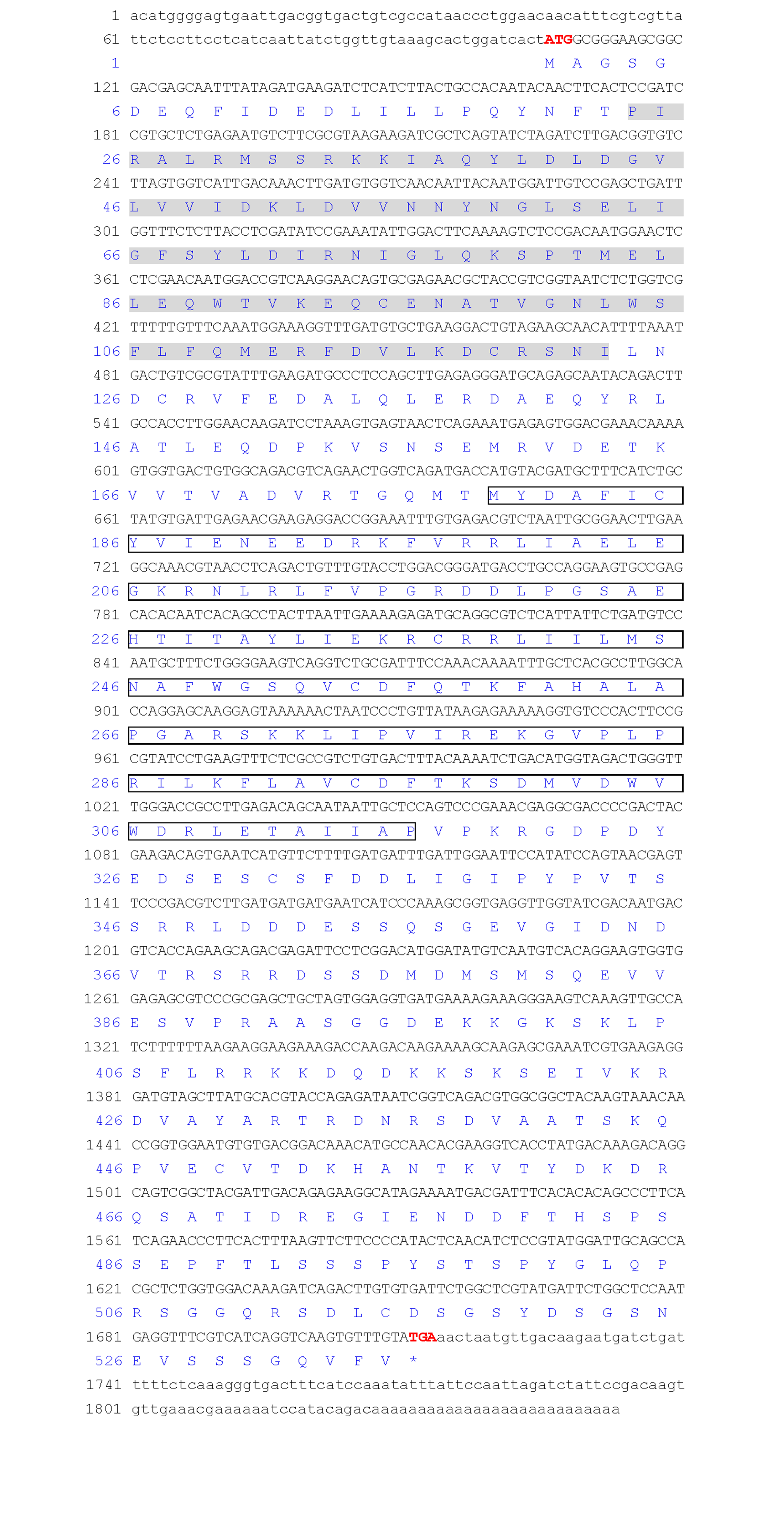

Supplement: S4 Fig — The predicted amino acids sequence. was shown below the nucleotide sequence. The start codon and stop codon were presented in red. The death domain and TIR domain were marked with gray shadow and black frame respectively. (TIF) [file pone.0231399.s004.tif]
